# C-NODDI: a constrained NODDI model for axonal density and orientation determinations in cerebral white matter

**DOI:** 10.3389/fneur.2023.1205426

**Published:** 2023-08-03

**Authors:** Maryam H. Alsameen, Zhaoyuan Gong, Wenshu Qian, Matthew Kiely, Curtis Triebswetter, Christopher M. Bergeron, Luis E. Cortina, Mary E. Faulkner, John P. Laporte, Mustapha Bouhrara

**Affiliations:** Laboratory of Clinical Investigation, National Institute on Aging, National Institutes of Health, Baltimore, MD, United States

**Keywords:** axonal density, white matter, NODDI, aging, MRI

## Abstract

**Purpose:**

Neurite orientation dispersion and density imaging (NODDI) provides measures of neurite density and dispersion through computation of the neurite density index (NDI) and the orientation dispersion index (ODI). However, NODDI overestimates the cerebrospinal fluid water fraction in white matter (WM) and provides physiologically unrealistic high NDI values. Furthermore, derived NDI values are echo-time (TE)-dependent. In this work, we propose a modification of NODDI, named constrained NODDI (C-NODDI), for NDI and ODI mapping in WM.

**Methods:**

Using NODDI and C-NODDI, we investigated age-related alterations in WM in a cohort of 58 cognitively unimpaired adults. Further, NDI values derived using NODDI or C-NODDI were correlated with the neurofilament light chain (NfL) concentration levels, a plasma biomarker of axonal degeneration. Finally, we investigated the TE dependence of NODDI or C-NODDI derived NDI and ODI.

**Results:**

ODI derived values using both approaches were virtually identical, exhibiting constant trends with age. Further, our results indicated a quadratic relationship between NDI and age suggesting that axonal maturation continues until middle age followed by a decrease. This quadratic association was notably significant in several WM regions using C-NODDI, while limited to a few regions using NODDI. Further, C-NODDI-NDI values exhibited a stronger correlation with NfL concentration levels as compared to NODDI-NDI, with lower NDI values corresponding to higher levels of NfL. Finally, we confirmed the previous finding that NDI estimation using NODDI was dependent on TE, while NDI derived values using C-NODDI exhibited lower sensitivity to TE in WM.

**Conclusion:**

C-NODDI provides a complementary method to NODDI for determination of NDI in white matter.

## Introduction

Postmortem histological investigations have shown that cerebral tissue undergoes continuous microstructural and architectural changes throughout the lifespan (1–3). It has been suggested that axonal degeneration is among the main sequelae of aging as well as several age-related disorders, with concomitant motor and cognitive declines (4–12). Therefore, it is indispensable to characterize changes in axonal density that occur with normative aging to identify alterations arising from pathological manifestations. Whilst providing insights into cerebral gray matter (GM) and white matter (WM) maturation and degeneration, histological investigations cannot be performed in real-time on living subjects precluding longitudinal evaluations of brain aging, correlative studies with physical and cognitive performance, or interventions.

Magnetic resonance imaging (MRI), particularly diffusion tensor imaging (DTI), has been extensively used to investigate brain maturation and degeneration, suggesting complex and nonlinear trajectories of the DTI-based indices with age in WM and GM (13–31). Although DTI-indices are sensitive to the fraction of intracellular water, a proxy of axonal density, they are also sensitive to other tissue properties, such as fiber crossing and fanning, while lacking specificity to different diffusion tissue compartments. To overcome this difficulty, the neurite orientation dispersion and density imaging (NODDI) MRI technique has been introduced, providing measures of neurite density and dispersion through computation of the orientation dispersion index (ODI) and the neurite density index (NDI) (32). NODDI has been extensively used in clinical and preclinical studies of aging, neurological disorders, and cognitive function (33–54). However, NODDI-based studies of axonal density and dispersion with normative aging remain limited, and with disparate results. Indeed, Billiet and colleagues and Chang and colleagues observed higher NDI values with age in several cerebral WM regions (43, 44), Merluzzi and colleagues’ observed lower NDI values in different cerebral WM structures (46), while Qian and colleagues, Lawrence and colleagues, and Beck and colleagues have recently shown a complex regional association between NDI and age, with several cerebral structures exhibiting inverted U-shaped relationships (6, 7, 55). These observations suggest an increase in axonal density until middle age followed by a loss afterwards (6, 7). It remains unclear whether this discrepancy is due to differences in cohort characteristics or the experimental implementation of NODDI, including variations in echo-time (TE) (56).

NODDI is based on a multicompartmental model of water diffusion incorporating intracellular water, that is, water within neurites, extracellular water, and a compartment consisting of less restricted water from the cerebrospinal fluid (CSF) volume. Although NODDI has gained rapid popularity, criticisms were raised for its overestimation of the isotropically diffusing water fraction (*f*_iso_) of the CSF compartment and for providing unrealistically high NDI values in WM (32, 56, 57). Newly, Gong and colleagues have shown that derived NDI values from NODDI are dependent on the echo time (TE) (56). These drawbacks, hampering result interpretation and precluding multisite comparisons, are believed to be due to the underlying assumption in the original NODDI signal model where all compartments are considered to have similar transverse relaxation (*T_2_*) values (56, 57). Indeed, in recent works, Bouyagoub and colleagues have suggested rescaling *f*_iso_ using predetermined *T_2_* values of the CSF and intra/extracellular water compartments (57), while Gong and colleagues proposed a multi-echo time NODDI (MTE-NODDI) approach incorporating several NODDI scans performed at different TEs (56). Although these compelling advanced approaches have led to plausible NDI values in WM, they require a lengthy extension of the total scan time making them hardly practical in clinical setting.

In this work, we propose a modification of NODDI that requires no extension of the acquisition time. Our approach is based on the modification of the NODDI signal model such that *f*_iso_ is provided as an input (i.e., constrained) value in each voxel. This bicomponent model simplifies the tricomponent model used in the original NODDI. We named this approach: constrained NODDI (C-NODDI). Unlike MTE-NODDI, C-NODDI assumes identical *T_2_* values for both the intra and extra-cellular waters, in line with Bouyagoub and colleagues’ original formulation (57). Indeed, this assumption is supported by extensive evidence from previous relaxometry studies, demonstrating that the relaxation times of these two compartments are close (58–61). Using the original NODDI and C-NODDI approaches, we investigated age and sex-related microstructural alterations in WM in a cohort of 58 cognitively unimpaired adults. Further, we compared the correlations between NDI derived values using NODDI or C-NODDI with the neurofilament light chain (NfL) concentration levels, a plasma biomarker of axonal degeneration (62–66), obtained from a subset of 43 participants included in our study cohort. Finally, we investigated the sensitivity of derived NDI and ODI values using NODDI or C-NODDI to TE in two participants from diffusion imaging data acquired at different TEs.

## Materials and methods

### Participants

**Figure 1:**
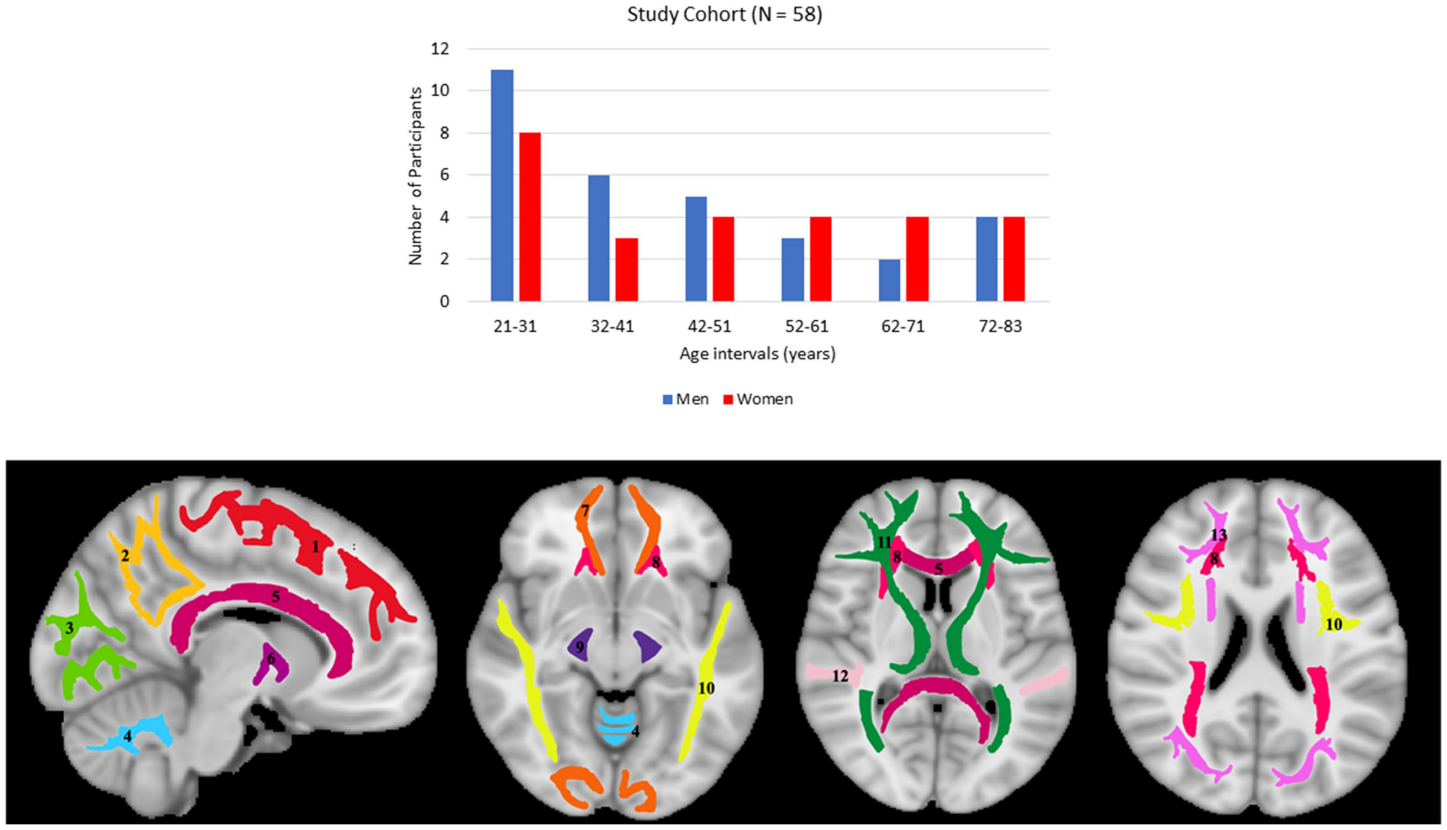
Upper panel: number of participants per age decade and sex within the study cohort. Lower panel: visualization of the white matter ROIs used in our analysis. (1) Frontal lobes (FL), (2) parietal lobes (PL), (3) occipital lobes (OL), (4) cerebellum (CRB), (5) corpus callosum (CC), (6) internal capsules (IC), (7) forceps (FR), (8) corona radiata (CR), (9) cerebral peduncles (CP), (10) longitudinal fasciculus (LF), (11) thalamic radiation (TR), (12) temporal lobes (TL), (13) fronto-occipital fasciculus (FOF).

Participants underwent a battery of cognitive tests and those with cognitive impairment, metallic implants, neurologic, or significant medical disorders were excluded (67). The final cohort consisted of 58 cognitively unimpaired volunteers (mean ± standard deviation of Mini-Mental State Examination (MMSE) = 29.2 ± 1.0) ranging in age from 21 to 83 years (45.4 ± 18.3 years), including 31 men (42.9 ± 17.5 years) and 27 women (48.3 ± 19.1 years). Age and MMSE did not differ significantly between men and women. The distribution of the number of participants per age decade and sex is shown in Figure 1. Experimental procedures were performed in compliance with our local Institutional Review Board, and participants provided written informed consent.

### Data acquisition

All experiments were performed with a 3T whole body Philips MRI system (Achieva, Best, The Netherlands) using the internal quadrature body coil for transmission and an eight-channel phased-array head coil for reception. Diffusion-weighted images (DWI) were acquired using a single-shot EPI sequence with repetition time (TR) of 10 s, echo time (TE) of 67 ms, three *b*-values of 0, 700, and 2000 s/mm^2^, with the two later encoded in 32 diffusion-weighting gradient directions, field-of-view (FoV) of 240 mm × 208 mm × 150 mm, acquisition matrix of 120 × 120 × 50, and acquisition voxel size of 2 mm × 2 mm × 3 mm. Images were acquired with SENSE factor of 2 and reconstructed to a voxel size of 2 mm × 2 mm × 2 mm. The total acquisition time was ~16 min.

Finally, to evaluate the effect of TE on NDI and ODI derived using NODDI or C-NODDI, DW images were acquired from the brains of two participants at four different TEs of 78, 90, 100 or 120 ms. Here again, three *b*-values of 0, 700, and 2000 s/mm^2^ were acquired, with the two later encoded in 32 diffusion-weighting gradient directions sampling the same *q*-space, FoV of 240 mm × 208 mm × 150 mm, acquisition matrix of 120 × 120 × 50, and acquisition voxel size of 2 mm × 2 mm × 3 mm.

### The C-NODDI signal model

#### Signal model

NODDI is a multicompartmental signal model of water diffusion incorporating intracellular water, extracellular water and CSF water volumes (32). Assuming that each of these three compartments exhibits unique transverse and longitudinal relaxation times and diffusion coefficient, the signal model can be expressed as:


(1) $$S=S_{0}[\begin{array}{l} f_{in}e^{-\frac{TE}{T_{2,in}}}(1-e^{-\frac{TR}{T_{1,in}}})S_{D,in}+f_{ex}e^{-\frac{TE}{T_{2,ex}}}(1-e^{-\frac{TR}{T_{1,ex}}})S_{D,ex}\\ +f_{iso}e^{-\frac{TE}{T_{2,iso}}}(1-e^{-\frac{TR}{T_{1,iso}}})S_{D,iso} \end{array}]$$

where S is the measured signal at a given combination of TE, repetition time (TR) and *b*-value; $S_{0}$ is the signal at TE = 0 ms with TR = +∞ and *b*-value = 0 s/mm^2^; $f_{in}$ , $f_{ex}$ and $f_{iso}$ are, respectively, the fractions of the intracellular, extracellular and CSF water pools; $T_{2,in}$ , $T_{2,ex}$ and $T_{2,iso}$ are, respectively, the transverse relaxation times of the intracellular, extracellular and CSF water pools; $T_{1,in}$ , $T_{1,ex}$ and $T_{1,iso}$ are, respectively, the longitudinal relaxation times of the intracellular, extracellular and CSF water pools; and $S_{D,in}$ , $S_{D,ex}$ and $S_{D,iso}$ are, respectively, the signal attenuation due to water diffusion of the intracellular, extracellular and CSF water pools. When long TR is applied (e.g., TR > > *T_1_*), the *T_1_* effect is mitigated so that Eq. 1 can be reduced to:


(2) $$\frac{S}{S_{0}}=f_{in}\;e^{-\frac{TE}{T_{2,in}}}\;S_{D,in}+f_{ex}\;e^{-\frac{TE}{T_{2,ex}}}\;S_{D,ex}+f_{iso}\;e^{-\frac{TE}{T_{2,iso}}}\;S_{D,iso}$$

Furthermore, it has previously been shown that $T_{2,in}\approx T_{2,ex}=T_{2,in/ex}$ (58–61), in which case Eq. 2 can be reduced to:


(3) $$A=f_{in}S_{D,in}+f_{ex}S_{D,ex}+F_{iso}S_{D,iso}$$

where $A=S/S_{b=0}$ is the measured normalized diffusion-weighted signal, with $S\mathrm{b=0}=S0\exp(-TE/T_{2,in/ex})$ representing the *T_2_*-weigthed image obtained at *b*-value of 0 s/mm^2^, and $F_{iso}\;=f_{iso}\;\exp(-TE/T_{2,iso}+TE/T_{2,in/ex}).$ In voxels with no CSF contamination such as the deep WM regions, $F_{iso}=0$ so that Eq. 3 can be reduced to $A=f_{in}S_{D,in}+\left(1-f_{in}\right)S_{D,ex}$ , in which case, derived NDI (i.e., $f_{in}$ ) values are expected to be relatively independent of *T_2_* and, therefore, are also relatively independent of the choice of TE. However, in regions with CSF contamination, i.e., $F_{iso}>0$ , which must be estimated.

It must be emphasized that C-NODDI relies on the strong assumption that the transverse relaxation times of the intracellular and extra-cellular waters exhibit similar values. However, separation in relaxation times between intracellular and extracellular water compartments remains an open area of investigation. While relaxometry-based studies consistently indicate minimal differences in these transverse relaxation times despite employing different acquisition strategies and fitting approaches (58–60, 68), some diffusion-relaxometry-based studies suggest the potential for discrepant values (56, 69).

#### NDI and ODI mapping

In the original NODDI approach, *F*_iso_ is derived along with NDI and ODI (32). We modified the NODDI MATLAB toolbox^1^ so that *F_iso_* is instead provided as a known input parameter. This strategy allows a reduction of the number of unknown parameters to be estimated (Eq. 3), thus restricting the parameter space and improving the fitting stability of the C-NODDI signal model (70–73). Here, the *F_iso_* map is computed from the *T_2_*-weigthed image obtained at *b*-value of 0 s/mm^2^ using the hidden Markov random field model and the expectation–maximization algorithm (74), known as FAST in the FSL software (75). FAST segments a structural image of the brain into different tissue classes, including WM, GM and CSF/*F_iso_*, providing, in each voxel, estimates of the fractions of these tissue compartments. Extensive work has previously been conducted to evaluate the accuracy of FAST for tissue segmentation (76–78). The derived *F*_iso_ map is then used as an input to calculate NDI and ODI using our modified NODDI approach, C-NODDI. All MATLAB codes are available upon request from the corresponding author.

For each participant, corresponding NDI and ODI maps were generated using NODDI and C-NODDI. Specifically, all DW images were registered to the *b_0_* image and corrected for motion and eddy current distortion artifacts using the Artefact Correction in Diffusion MRI (ACID) toolbox^2^ (79). The co-registered DW images were then used to calculate NDI and ODI using NODDI or C-NODDI.

### Regions-of-interest determination

For each participant, using FSL, the DW image obtained with *b* of 0 s/mm^2^ was nonlinearly registered to the Montreal Neurological Institute (MNI) standard space and the derived transformation matrix was then applied to the corresponding NDI and ODI maps. Fourteen WM regions of interest (ROIs) were defined from MNI encompassing the whole brain (WB), frontal lobes (FL), parietal lobes (PL), temporal lobes (TL), occipital lobes (OL), cerebellum (CRB), corpus callosum (CC), internal capsules (IC), cerebral peduncle (CP), corona radiata (CR), thalamic radiation (TR), fronto-occipital fasciculus (FOF), longitudinal fasciculus (LF), and forceps (FR), as shown in Figure 1. All ROIs were eroded to reduce partial volume effects and imperfect image registration using the FSL tool *fslmaths.* Finally, the mean NDI and ODI values within each ROI were calculated.

### Analyses

#### Differences in *F_iso_*

In this analysis, we compared derived *F_iso_* maps using NODDI or FAST. Derived *F_iso_* maps were shown for a representative example. The goal here is to demonstrate the overestimation of *F_iso_* from NODDI, and to evaluate the performance of FAST in *F_iso_* determination.

#### Differences in NDI and ODI

In this analysis, we compared NDI and ODI maps derived using NODDI or C-NODDI. Average NDI and ODI maps by age intervals over the adult lifespan for a representative axial slice were calculated. Furthermore, to investigate the effects of age and sex on NDI and ODI, multiple linear regression analysis was applied using the mean NDI or ODI derived from NODDI or C-NODDI within each ROI as the dependent variable and sex, age, and age^2^ as the independent variables, after mean age centering. In all cases, the interactions between sex and age or age^2^ were found to be nonsignificant and were therefore omitted from the parsimonious model. Further, for each ROI, Pearson correlation analysis was conducted to examine the discrepancy between ODI, or NDI, values derived using NODDI or C-NODDI. For all analyses, the threshold for statistical significance was *p* < 0.05 after correction for multiple ROI comparisons using the FDR method (80, 81). FDR correction was conducted across ROIs for each MRI metric.

#### Correlations of NDI and NfL

In this analysis, we assessed the correlations between NDI derived using NODDI or C-NODDI and NfL which represents a plasma biomarker of axonal degeneration (62–66). NfL was measured from 43 participants of our study cohort (age: 50 [SD 18]). Blood for plasma biomarker measurement was collected at the time of MRI. Plasma was separated, aliquoted and stored at −80°C using standardized protocols. EDTA plasma was used to measure NfL using the Quanterix Single Molecule Array (Simoa) Neurology 4-Plex E assay on the HD-X Instrument (Quanterix Corporation). NfL was used as the dependent variable and NDI as the independent variables while accounting for age and sex as relevant covariates. The regression model is given by:


$${\mathrm{NFL}}_{i}\sim \beta_{0}+\beta_{NDI}\times {\mathrm{NDI}}_{i}+\beta_{age}\times {\mathrm{age}}_{i}+\beta_{sex}\times {\mathrm{sex}}_{i},$$

where β is the regression coefficients and *i* is the index of *i*^th^ subject. NfL values were log-transformed to remove the skewness of their distributions. Further, to facilitate results interpretation, the NfL and NDI indices were Z-scored by calculating the mean (μ) and standard deviation (σ) separately for NfL and NDI variables in the full study sample using the formula z = (x – μ)/σ.

#### Effect of TE

To investigate the effect of TE on derived NDI and ODI values using C-NODDI or NODDI, parameter maps as well as mean values calculated over a large WM region encompassing the cerebral lobes, at each TE, were displayed for two participants.

## Results

**Figure 2:**
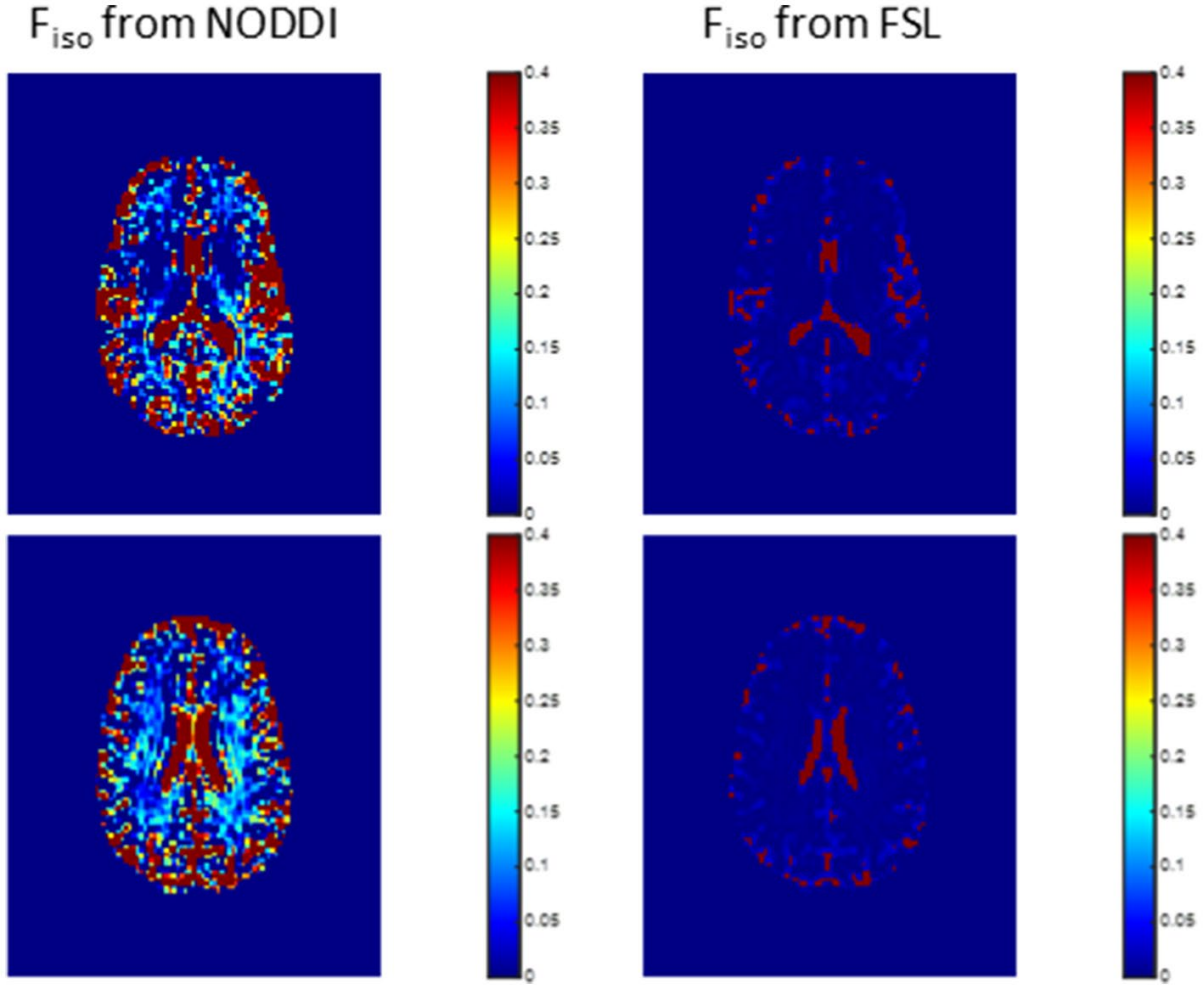
Representative maps of the fraction of the isotropic diffusion component *F_iso_*, that is, the cerebrospinal fluid fraction, derived using NODDI (left) or using the FAST algorithm as implemented in the FSL software (right). Results are shown for three representative slices. It is readily seen that the NODDI approach overestimates *F*_iso_, especially in the white matter regions.

Figure 2 shows representative *F_iso_* maps derived using NODDI or FAST/FSL. It is readily seen that th *F_iso_* map derived using NODDI exhibits abnormally high *F_iso_* values, especially within several WM regions, with values reaching over 0.25 (i.e., 25%) in several voxels. In contrast, derived *F_iso_* values using the hidden Markov random field model and the expectation–maximization algorithm, the FAST algorithm as implemented in FSL, are within the physiologically expected ranges with values near zero in the deep WM regions.

**Figure 3:**
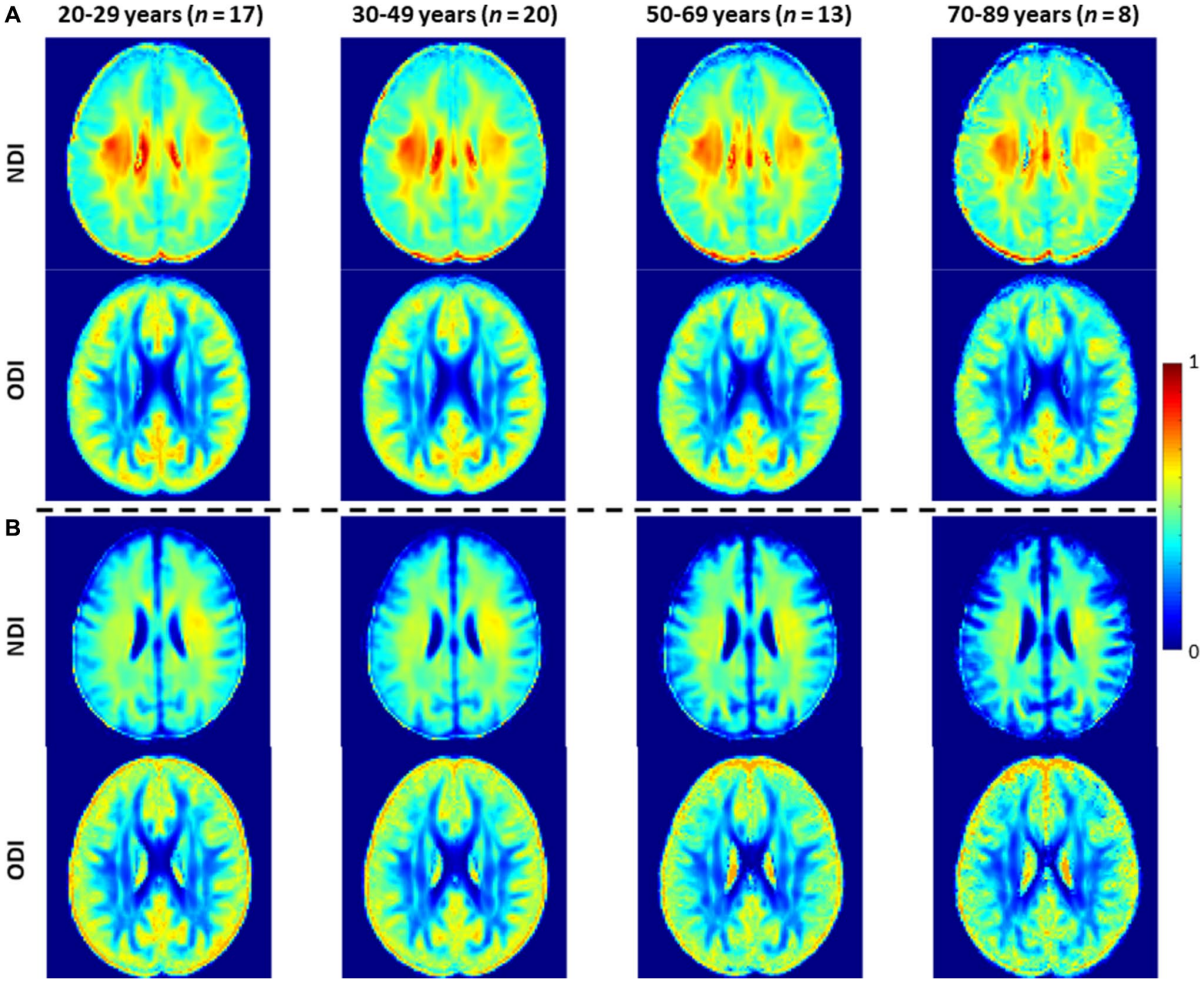
NDI and ODI maps represented as averaged participant maps calculated over four age intervals. Results are shown for a representative slice. NDI and ODI maps derived **(A)** using the original NODDI approach, and **(B)** using the C-NODDI approach. While the ODI values derived using both approaches are virtually identical, C-NODDI provides substantially lower NDI values than those derived using NODDI.

**Figure 4:**
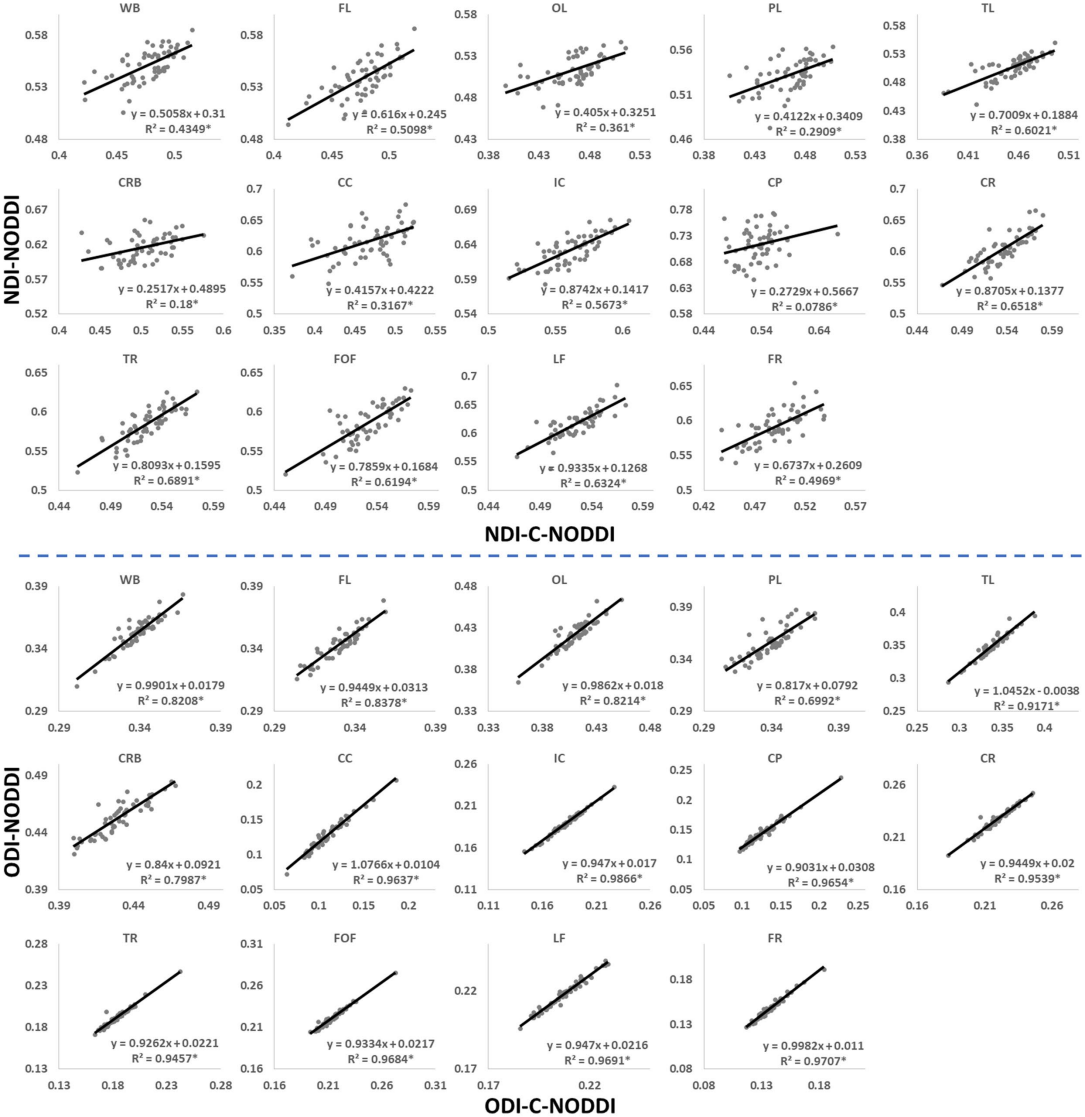
Pearson correlations between NDI, or ODI, derived values using C-NODDI and NODDI in the 14 WM ROIs studied. The coefficient of determination is provided with * indicating significant correlation. While derived ODI values using NODDI or C-NODDI were virtually identical exhibiting very high correlation coefficients, the NDI values derived using these two approaches were substantially different with NODDI exhibiting higher NDI values in several WM regions. WB, whole brain; FL, frontal lobes; PL, parietal lobes; TL, temporal lobes; OL, occipital lobes; CRB, cerebellum; CC, corpus callosum; IC, internal capsules; CP, cerebral peduncle; CR, corona radiata; TR, thalamic radiation; FOF, fronto-occipital fasciculus; LF, longitudinal fasciculus; FR, forceps.

Figure 3 shows representative average NDI and ODI maps for different age intervals corresponding to young, middle, late middle and late adulthood. Visual inspection indicates increases in NDI values from early adulthood through middle age (i.e., 40–59 years), followed by lower NDI values in several brain regions, consistent with progressive increases in axonal density followed by reductions at older ages. Furthermore, we note that different regions exhibit different patterns in the association of NDI with age. In contrast, the ODI maps exhibit low regional variations with age. Remarkably, NDI maps derived from NODDI exhibit values that exceed 0.7 (i.e., 70%) in several cerebral WM structures, while the NDI values derived using C-NODDI are considerably lower. Moreover, ODI maps derived using NODDI and C-NODDI were virtually identical. Indeed, our quantitative comparison, presented in Figure 4, indicates weak to moderate regional Pearson correlation between the NDI values derived using NODDI and those derived using C-NODDI for all ROIs investigated, in agreement with visual inspection (Figure 3). In contrast, strong regional correlations were observed between the ODI values derived from the two NODDI approaches.

**Figure 5:**
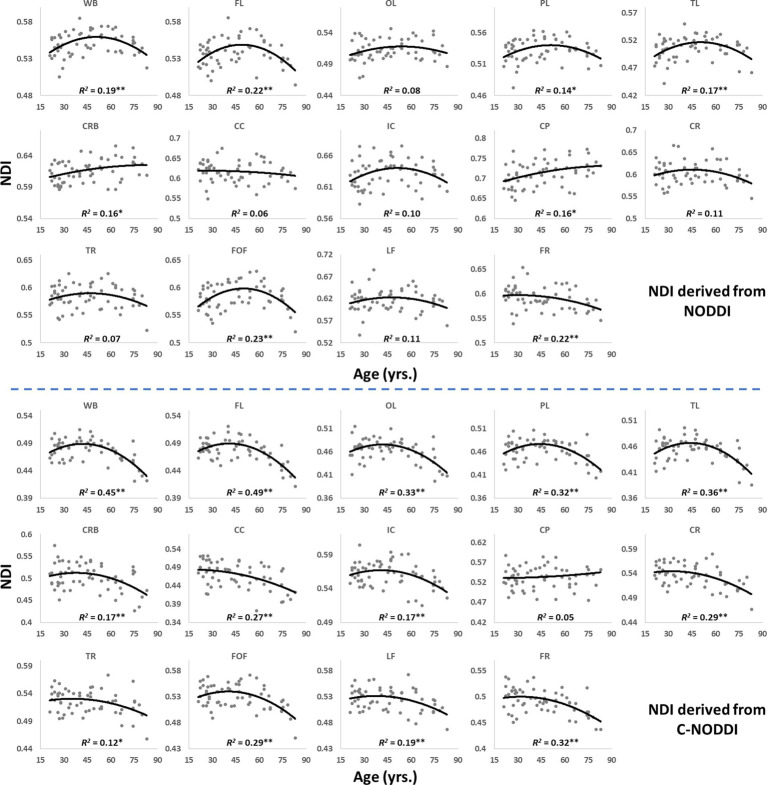
Regional NDI values derived using NODDI (top plots) or C-NODDI (bottom plots) as a function of age. Although most regions investigated exhibit inverted U-shaped trends of NDI with age, these quadratic trends were more noticeable and significant for the NDI-C-NODDI results (see Table 1). WB, whole brain; FL, frontal lobes; PL, parietal lobes; TL: temporal lobes; OL, occipital lobes; CRB, cerebellum; CC, corpus callosum; IC, internal capsules; CP, cerebral peduncle; CR, corona radiata; TR, thalamic radiation; FOF, fronto-occipital fasciculus; LF, longitudinal fasciculus; FR, forceps. * indicates *p* < 0.05 while ** indicates *p* < 0.01.

**Figure 6:**
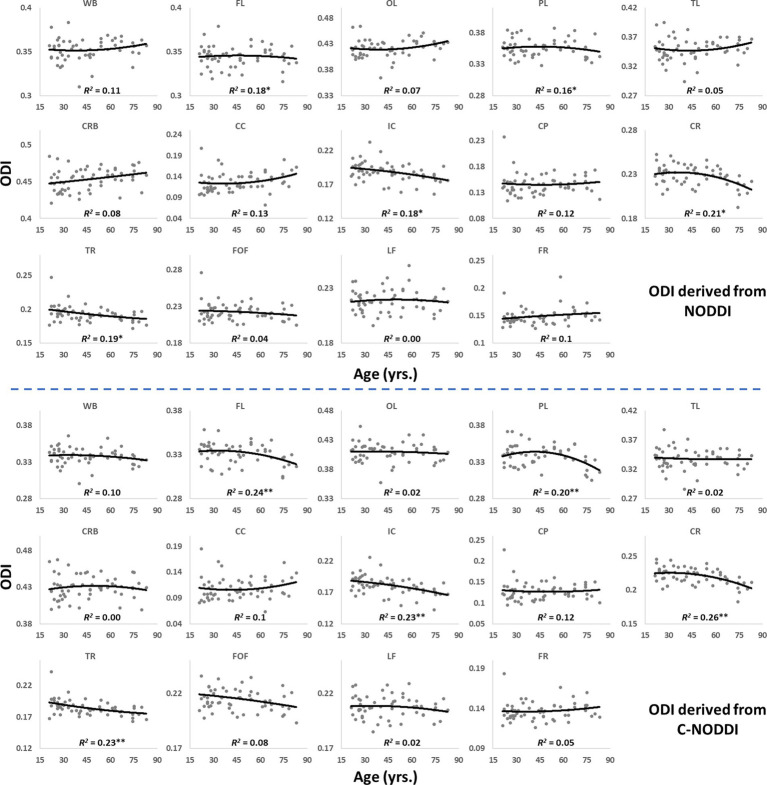
Regional ODI values derived using NODDI (top plots) or C-NODDI (bottom plots) as a function of age. Results were very similar for both approaches. WB, whole brain; FL, frontal lobes; PL, parietal lobes; TL, temporal lobes; OL, occipital lobes; CRB, cerebellum; CC, corpus callosum; IC, internal capsules; CP, cerebral peduncle; CR, corona radiata; TR, thalamic radiation; FOF, fronto-occipital fasciculus; LF, longitudinal fasciculus; FR, forceps.

Figures 5, 6 show, respectively, quantitative results for the NDI and ODI values calculated using NODDI or C-NODDI from all participants as a function of age for the indicated 14 WM regions. These results show, in agreement with Figures 3 4, increasing NDI until middle age followed by decreases afterward in most examined ROIs, with the best-fit curves displaying regional variation. Remarkably, the quadratic effect of age on NDI derived using C-NODDI was readily observable in almost all ROIs, as compared to the NDI results derived using NODDI which was limited to a few. Furthermore, in agreement with Figures 3 4, ODI values derived using either NODDI approach exhibited similar regional trends that were, overall, constant with age with limited ROIs exhibiting either increasing or decreasing trends. In agreement with visual inspection, statistical analysis indicates that the quadratic effect of age, age^2^, on NDI derived using C-NODDI was significant (*p* < 0.05) in several ROIs, while this effect on NDI derived using NODDI was limited to a few ROIs (Table 1). For both NODDI approaches, the quadratic effect of age on ODI was not significant. In contrast, the effect of age on NDI-C-NODDI was significant in almost all ROIs, while the age effect on NDI-NODDI was observed in only three ROIs. For both NODDI approaches, the effect of age on ODI was only significant in very limited cerebral regions. In addition, the effect of sex on NDI or ODI was, overall, not significant. Finally, in all ROIs, the NDI-C-NODDI results exhibited peak values at younger ages, as compared to the NDI results derived using NODDI.

**Table 1:** Significance of each coefficient incorporated into the linear regression analysis of NDI or ODI derived using NODDI or C-NODDI, and the year of apparent maximum NDI in each ROI.

	NODDI							C-NODDI						
	NDI				ODI			NDI				ODI		
	Age	Sex	Age^2^	Year of max NDI	Age	Sex	Age^2^	Age	Sex	Age^2^	Year of max NDI	Age	Sex	Age^2^
WB	–	–	**	50.8	–	–	–	**	–	**	42.3	–	–	–
FL	–	–	**	48.9	–	*	–	**	–	**	41.1	*	–	–
OL	–	–	–	NA	–	–	–	**	–	**	41.8	–	–	–
PL	–	–	*	50.5	–	*	–	**	–	**	44.6	*	–	–
TL	–	–	*	50.3	–	–	–	**	–	**	44.0	–	–	–
CRB	*	–	–	NA	–	–	–	*	–	–	NA	–	–	–
CC	–	–	–	NA	–	–	–	**	–	–	NA	–	–	–
IC	–	–	–	NA	*	–	–	*	–	–	NA	**	–	–
CP	*	–	–	NA	–	–	–	-	–	–	NA	–	–	–
CR	–	–	–	NA	*	–	–	**	–	–	NA	**	–	–
TR	–	–	–	NA	*	–	–	*	–	–	NA	**	–	–
FOF	–	–	**	49.9	–	–	–	**	–	*	40.6	–	–	–
LF	–	–	–	NA	–	–	–	**	–	–	NA	–	–	–
FR	*	–	–	NA	–	–	–	**	–	–	NA	–	–	–

NA indicates “not applicable” for a nonsignificant age^2^ term. * Indicates *p* < 0.05, ** indicates *p* < 0.01, and – indicates non-significant effects. All *p*-values presented are obtained after FDR correction. WB, whole brain; FL, frontal lobes; PL, parietal lobes; TL, temporal lobes; OL, occipital lobes; CRB, cerebellum; CC, corpus callosum; IC, internal capsules; CP, cerebral peduncle; CR, corona radiata; TR, thalamic radiation; FOF, fronto-occipital fasciculus; LF, longitudinal fasciculus; FR, forceps.

**Figure 7:**
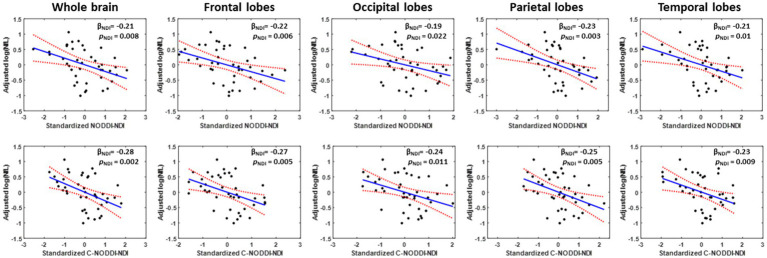
Representative correlation plots between neurodegeneration biomarker, NfL, and NDI values derived using NODDI (left plot) or C-NODDI (right plot) from the whole brain, frontal lobes, occipital lobes, parietal lobes, or temporal lobes. Line of fit (in blue), confidence interval lines (red), and the regression coefficient (β_NDI_) and significance (p_NDI_) of the NDI variable were displayed.

Our multiple regression between NDI and NfL indicates stronger correlations between NDI-C-NODDI vs. NfL as compared to NDI-NODDI vs. NfL, with lower NDI values corresponding to higher NfL concentration levels in all ROIs investigated (Figure 7). We also note that the negative trend between NDI and NfL was observed in all brain regions except in the cerebral peduncles (data not shown).

**Figure 8:**
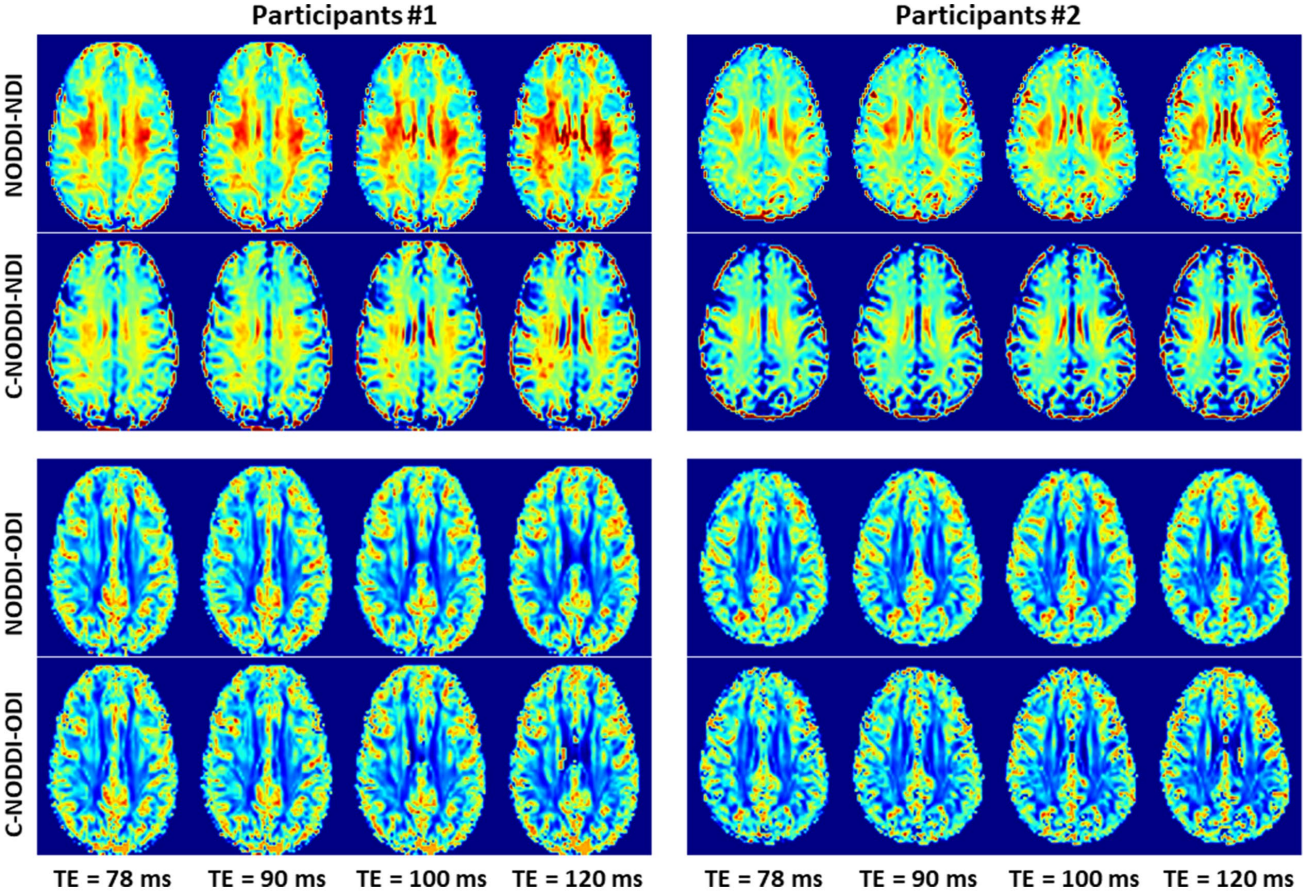
NDI and ODI maps derived using NODDI or C-NODDI from DWI data acquired at different TEs from the brains of two different participants. Results are shown for a representative slice for each of the participants.

Finally, Figures 8 9 show, respectively, examples of derived NDI and ODI maps and mean parameter values calculated over the whole WM region using NODDI or C-NODDI at several TEs using DWI data acquired from the brains of two participants. Visual inspection and quantitative analysis indicate that derived NDI estimates using NODDI are TE-dependent exhibiting increased values with TE increases. In contrast, the NDI maps and mean values calculated using C-NODDI exhibit lower dependence on TE. However, although the majority of the white matter regions exhibited relatively lower dependence on TE, some regions showed substantially increased values with TE. Further, it is readily seen that the ODI maps calculated using NODDI or C-NODDI are similar and with mean values virtually constant as a function of TE. Moreover, we note that derived NDI and ODI maps exhibit some regional differences across TEs; this is due to differences in signal-to-noise ratio due to differences in TEs leading to signal drops at higher TEs, as well as to some persisting image misregistration.

**Figure 9:**
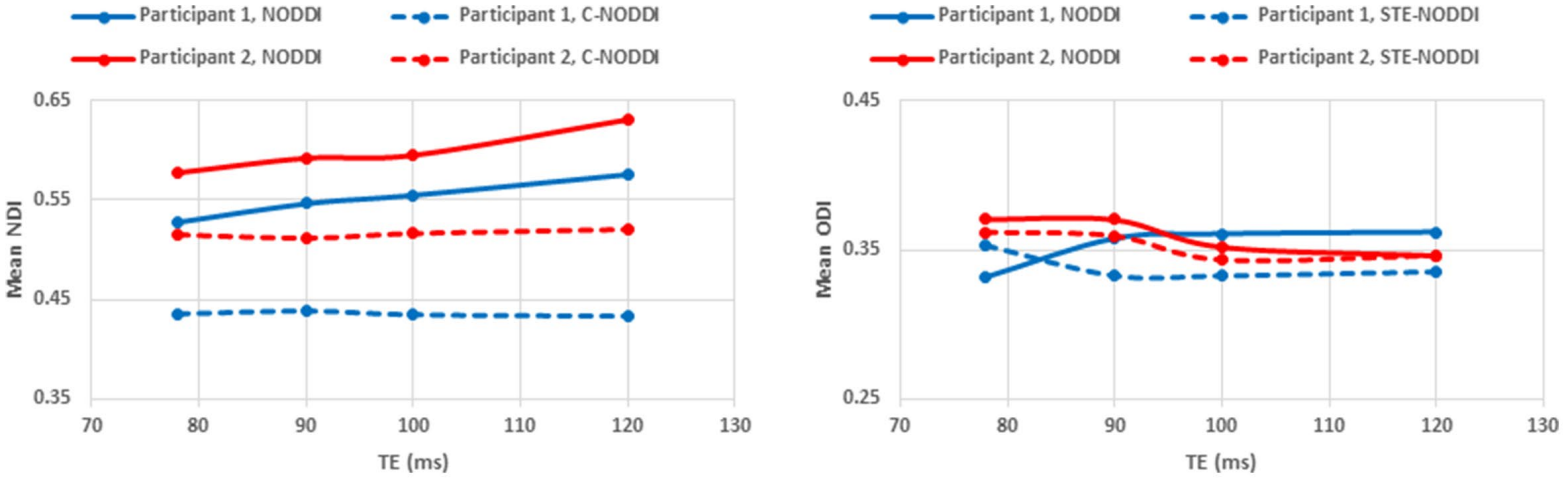
Mean NDI values and ODI values calculated from a large WM region encompassing the cerebral lobes, derived using NODDI or C-NODDI as a function of TE. It is readily seen from this quantitative analysis that the NDI values derived using NODDI increase with TE, while derived ODI values using either approach are relatively similar between both approaches with, overall, constant or decreasing trends as a function of TE, in agreement with the visual inspection (Figure 8).

**Figure 10:**
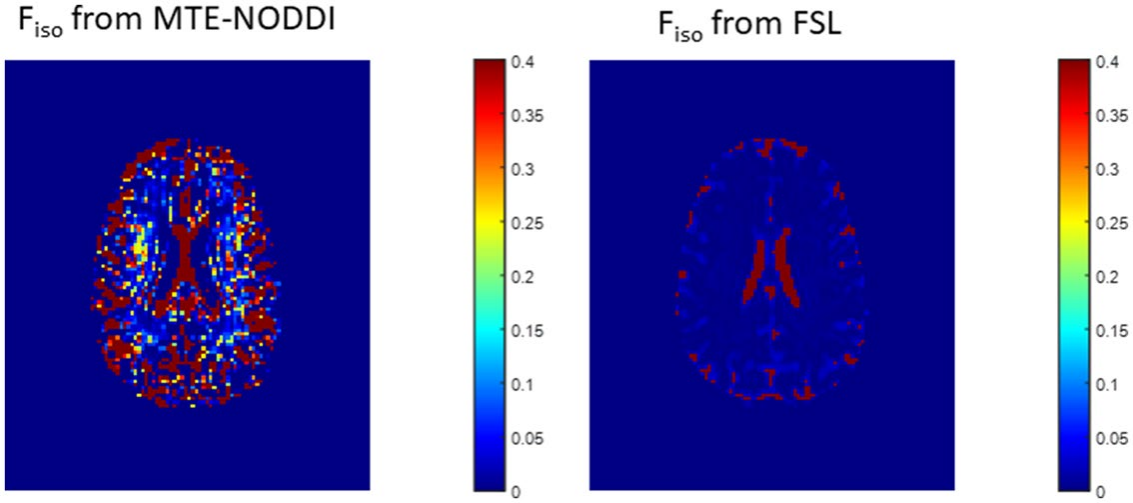
Representative maps of the fraction of the isotropic diffusion component (*F*_iso_), that is, the cerebrospinal fluid fraction, derived using MTE-NODDI (left) or using the FAST algorithm as implemented in the FSL software (right). Results are shown for a representative slice.

## Discussion and conclusion

In this work, we introduced a modification of the NODDI signal model, C-NODDI, to overcome documented problems with the original NODDI approach. Specifically, this modification addresses the overestimations of the CSF and NDI fractions in WM. Our results show that C-NODDI provides lower NDI estimates as compared to NODDI and confirm that NODDI provides high NDI values in several WM structures with values over 70% at several cerebral structures in agreement with previous reports (56, 57). NDI values derived from C-NODDI were regionally 20–40% lower than those derived using NODDI; this agrees with Bouyagoub and colleagues’ results (57). However, unlike their approach which requires a substantial extension of the total acquisition time, our approach does not require additional acquisition time. Indeed, C-NODDI is based on prior estimation of the signal fraction of the CSF. In this proof-of-concept work, we showed that this fraction can be estimated using the *T_2_*-weigthed image acquired at *b*-value of 0 s/mm^2^ and the hidden Markov random field model and the expectation–maximization algorithm; this allows reduction of the fitting parameter space of the NODDI model (74). Indeed, the NODDI signal model incorporates several parameters to be estimated. Parameter estimation in high dimensional problems, that is, those for which the number of parameters to be estimated is large, is complicated by the presence of local minima and saddle points (73). This problem becomes more acute with the flatness of the least-squares residual surfaces seen with increasing model complexity exhibiting higher sloppiness, that is, different parameter combinations leading to virtually equivalent signal behavior (70–72). However, both NODDI and C-NODDI signal models are just approximations of the underlying biology so that further histological validations are required to assess their reliability and accuracy. We note that the CSF fraction can be estimated using different approaches, including the free-water estimation (FWE) diffusion approaches from the same NODDI dataset, or using FAST from various other structural contrast-weighted images that are routinely acquired in clinical studies (82, 83), or from single shell diffusion (82, 84, 85). A thorough comparison of all these techniques is still needed, especially in the context of their applications to neurodegeneration. Nevertheless, the existing FWE diffusion approaches provide only an estimate of the apparent free water fraction due to the impact of the differential *T*_2_ weighting of the intra/extra and CSF/free water compartments. While MTE-NODDI provides CSF fraction maps that are TE-independent and with values that are relatively lower than those derived using NODDI (Figure 10), their determination is still based on the *F*_iso_ maps derived using NODDI at each TEs. Therefore, further investigations are needed to assess its reliability. Finally, we note that C-NODDI is in principle similar to a recently introduced technique, DLpN, where the CSF is used as a prior (84). A direct comparison between DLpN and C-NODDI as well as the association of derived NDI values with NfL is of interest and represent a potential direction of the current work.

We investigated the association between NDI or ODI, derived using NODDI or C-NODDI, and age in several cerebral WM structures in a healthy adult population spanning a wide age range. Our results revealed widespread WM microstructural differences as a function of age, as well as regional variations between the NDI or ODI measures and age. Specifically, NDI exhibited quadratic, inverted U-shaped, regional trends with age; this agrees with Beck and colleagues’ and our recent results (6, 7) and to some extent with Cox and colleagues’ and Laurence and colleagues’ observation in a large sample sizes derived from the UK Biobank (55, 86). We attribute this finding to a continuous maturation of axons with an increase in axonal density until middle age, followed by a phase of neurodegeneration and consequence axonal loss at older ages. Interestingly, the quadratic effect of age, age^2^, on NDI was significant (*p* < 0.05) in several regions investigated using C-NODDI, while this inverted U-shaped association was limited to only a few cerebral regions using the original NODDI approach. Although this observation indicates the potential higher sensitivity of C-NODDI to capturing differences in neurite density with age, further comparison between NODDI and C-NODDI in larger cohorts is still required to confirm or infirm this finding. We note that other investigations have shown either higher or lower NDI values with age in WM (43, 44, 46). This discrepancy is likely due to differences in the characteristics of the study cohorts as well the instability of NODDI. Indeed, Billiet’s and Chang’s studies, although incorporating wide age ranges, possess only a limited number of subjects over 60 years old, while Merluzzi’s cohort included only subjects over 45 years old. These limitations may have precluded the detection of the quadratic association between NDI and age, that Beck, Lawrence and we have observed (6, 7, 55). This issue may have been exacerbated by differences in the experimental implementation of NODDI given its high sensitivity to TE (56). Further, ODI exhibited non-consistent regional trends with age, with most WM regions exhibiting constant trends while other, but limited, regions exhibited either increasing or decreasing trends. The literature regarding differences in ODI with age remains sparse, necessitating further detailed investigations (6, 43, 44, 46). Finally, our results revealed that elevated concentration levels of NfL were associated with lower NDI values suggesting a role of axonal degeneration in neuroinflammation. This association was stronger between NDI derived using C-NODDI and NfL, suggesting that NDI-C-NODDI could represent a reliable imaging biomarker of axonal integrity. Further studies in larger cohorts and using NfL concentrations derived from CSF are still required.

In all ROIs, NDI calculated using C-NODDI exhibited peak values at much younger ages compared to NDI calculated using NODDI, with differences ranging from ~6 to ~10 years. This indicates that axonal maturation continues until the early fifth decade of age. This finding agrees with results derived using sensitive MRI measures of axonal density, including relaxation times and DTI indices, all indicating that WM tissue maturation continues until the late fourth decade to the early fifth decade of age (15, 16, 18, 87). Interestingly, recent studies have shown that myelination continues until the early fifth decade of age as well (17, 88, 89). It has been shown that axonal activity is an important contributing factor in myelin modulation (90, 91). Moreover, aside from acting as an electric insulator, oligodendrocytes, the cells that produce myelin, provide substantial metabolic support to axons (92). Given this physiological coupling between axons and myelination, it is plausible that axonal maturation and myelin maturation follow similar patterns and peak at similar ages. However, further investigations on larger cohorts, in addition to longitudinal studies, are required to derive definite conclusions regarding whether the maturation and myelination pattern of axons are mechanistically associated or represent two independent neurological processes. These studies are crucial for the development of specific interventions supporting myelin maintenance, axonal regeneration, or both simultaneously.

Using data acquired from three participants at different TEs, we confirmed Gong and colleagues’ observation that the NDI estimates are dependent on TE, with NDI values increasing with TE (56). However, derived NDI values using C-NODDI exhibited a relatively lower dependence on TE in several WM structures. We believe that the artificial overestimation of the CSF fraction using NODDI is a leading factor of the strong underlying TE-dependency observed previously, particularly in WM. It is important to note that certain WM regions still exhibited TE-dependency in C-NOODI, with relatively higher values observed at increasing TE. Thus, further examination of the effect of T_2_s on derived diffusion parameters is required as well as a comprehensive comparison of these approaches under different experimental designs, including in terms of different ranges and numbers of TEs and *b*-values. Furthermore, in perfect agreement with Bouyagoub’s and Gong’s results (56, 57), we found that ODI values derived using NODDI or C-NODDI were virtually identical, as expected. Since the calculation of ODI does not incorporate signal fractions, it remains insensitive to differences in the CSF variation.

There are various techniques available to estimate NDI or ODI from single-shell or multishell DTI data (56, 69, 93–95). One of these techniques is the NODDI-DTI which assumes null CSF fractions in the WM (95). This contrasts with C-NODDI that uses the isotropic water fraction as a known pre-determined parameter. Interestingly, unlike C-NODDI, NODDI-DTI provides NDI values that are similar to those derived using NODDI. However, NODDI-DTI provides a unique approach to approximate NDI values from single-shell DTI, which has been used before by our group to reduce the scan time needed for whole-brain aggregate g-ratio mapping (30). However, it is important to acknowledge that all these methods are based on model simplifications, biophysical assumptions, fixed parameters, or complex fitting approaches, which may lack validation, stability, or clinical practicality (96, 97). However, there is a great deal of research currently toward further validation of these assumptions especially in preclinical research for more accurate determination of NDI or ODI. While challenging, more work must be conducted to make these techniques truly translatable to clinical investigations (98, 99). We believe that our approach introduced here for physiologically plausible NDI value and the validation against NfL opens the way for further developments to improve the NODDI modeling.

Our work has limitations. Although our cohort spans a wide age range, it does not include participants under the age of 20 due to the inclusion and exclusion criteria of the BLSA and GESTALT studies. Including younger participants may influence the shape of the NDI or ODI age-related trends and the assessment of their respective maxima with respect to age (100). With our current dataset being cross-sectional, our results could be further validated through longitudinal studies; such work is underway. In addition, the NODDI and C-NODDI implementations are based on several assumptions and fixed values for certain diffusivity parameters to improve the stability of the signal model. Further analyses are needed to investigate the effects of these fixed parameters on the NODDI and C-NODDI outcomes. Moreover, our acquisition protocol included only one image at *b*-value of 0 s/mm^2^. However, it has been shown that several *b_0_* images are critical for an accurate determination of diffusion parameters (101). We also note that NODDI and C-NODDI assume similar transverse relaxation times between the intracellular and extracellular water compartments. This assumption is based on extensive relaxometry-based as well as diffusion-relaxometry-based work (58–61, 102). Although our results showing lower dependence of TE for C-NODDI derived NDI values in different brain regions support our assumption of relatively similar T_2_s between the intra- and extra-cellular water compartments, other regions exhibit dependence to TE in agreement with previous work suggesting that these compartments exhibit different *T*_2_s (56, 103). While our investigation has not yielded a definitive conclusion on this assumption, this observation highlights the complexity of brain microstructure and the potential limitations of a “one assumption fits all” approach given the fact that all these methods are simplifications based on biophysical assumptions that are often not validated or fitting approaches that are unstable which could lead to spurious results. We remain dedicated to further exploring this intriguing discrepancy within the existing literature. Finally, our method was applied to cognitively unimpaired individuals with further investigations required in the context of neurodegeneration and cognitive impairment. This is a proof-of-concept study, and further validation is warranted by acquiring data from different sites and scanners, as well as through post-mortem studies.

## Data availability statement

The raw data supporting the conclusions of this article will be made available by the authors, without undue reservation.

## Ethics statement

The studies involving human participants were reviewed and approved by the NIA ethics committee IRB. The patients/participants provided their written informed consent to participate in this study.

## Author contributions

MB: theoretical and experimental design, data acquisition, results interpretation, and manuscript writing and editing. MA and ZG: data acquisition and analysis, results interpretation, and manuscript writing and editing. MK, CT, LC, MF, and JL: data analysis, results interpretation, and editing. CB: data acquisition and manuscript editing. All authors contributed to the article and approved the submitted version.

## Conflict of interest

The authors declare that the research was conducted in the absence of any commercial or financial relationships that could be construed as a potential conflict of interest.

## Publisher’s note

All claims expressed in this article are solely those of the authors and do not necessarily represent those of their affiliated organizations, or those of the publisher, the editors and the reviewers. Any product that may be evaluated in this article, or claim that may be made by its manufacturer, is not guaranteed or endorsed by the publisher.

## Footnotes

^1^ https://www.nitrc.org/projects/noddi_toolbox

^2^ http://diffusiontools.com/
